# Supplementary material for: Sorafenib Resistance Contributed by IL7 and MAL2 in Hepatocellular Carcinoma Can Be Overcome by Autophagy-Inducing Stapled Peptides
Source: Cancers (Basel). 2023 Nov 3;15(21):5280. doi: 10.3390/cancers15215280 (PMC10650575; doi:10.3390/cancers15215280)
Supplement: Supplementary file 1 [file cancers-15-05280-s001.zip › Supplementary data/Supplymentary figure S1.pdf]

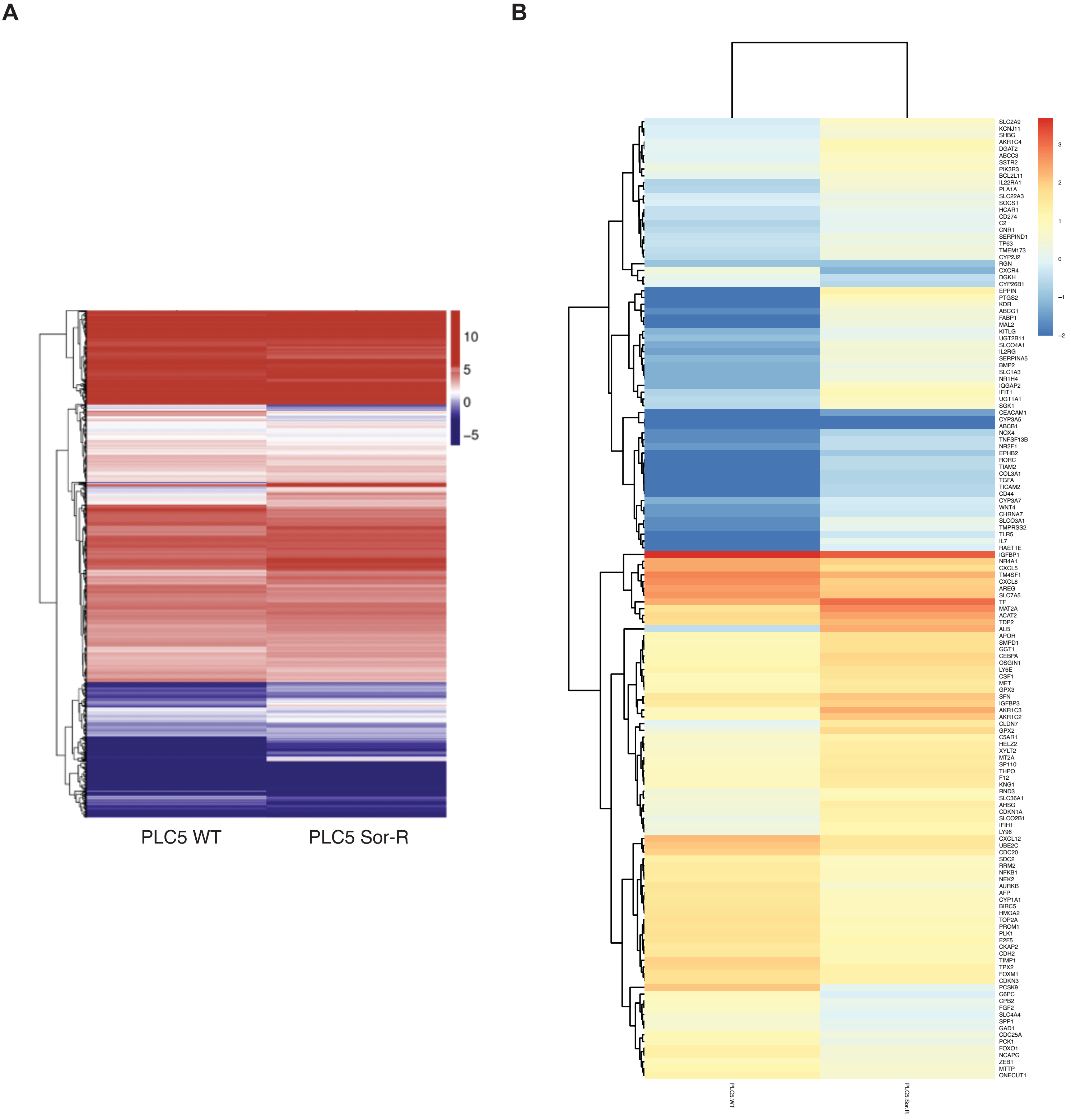

Supplementary Figure S1 Identification of *IL7* and *MAL2* as candidate genes in HCC-associated Sorafenib resis-tance. (**A**) RNA-seq heatmap of 3,255 differentially expressed genes (DEGs) showing differences in gene expression profiles between PLC/PRF/5 wild-type (PLC5 WT) and Sorafenib resistant mutant (PLC5 Sor-R) cells – 2,240 genes were upregulated and 1,015 genes were downregulated. (**B**) Under further stringent selection criteria (P-value < 0.01, FDR < 0.02, Fold-change: ± 1.5), 142 candidate genes were identified in contributing to Sorafenib resistance – 96 upregulated genes and 46 downregulated genes.

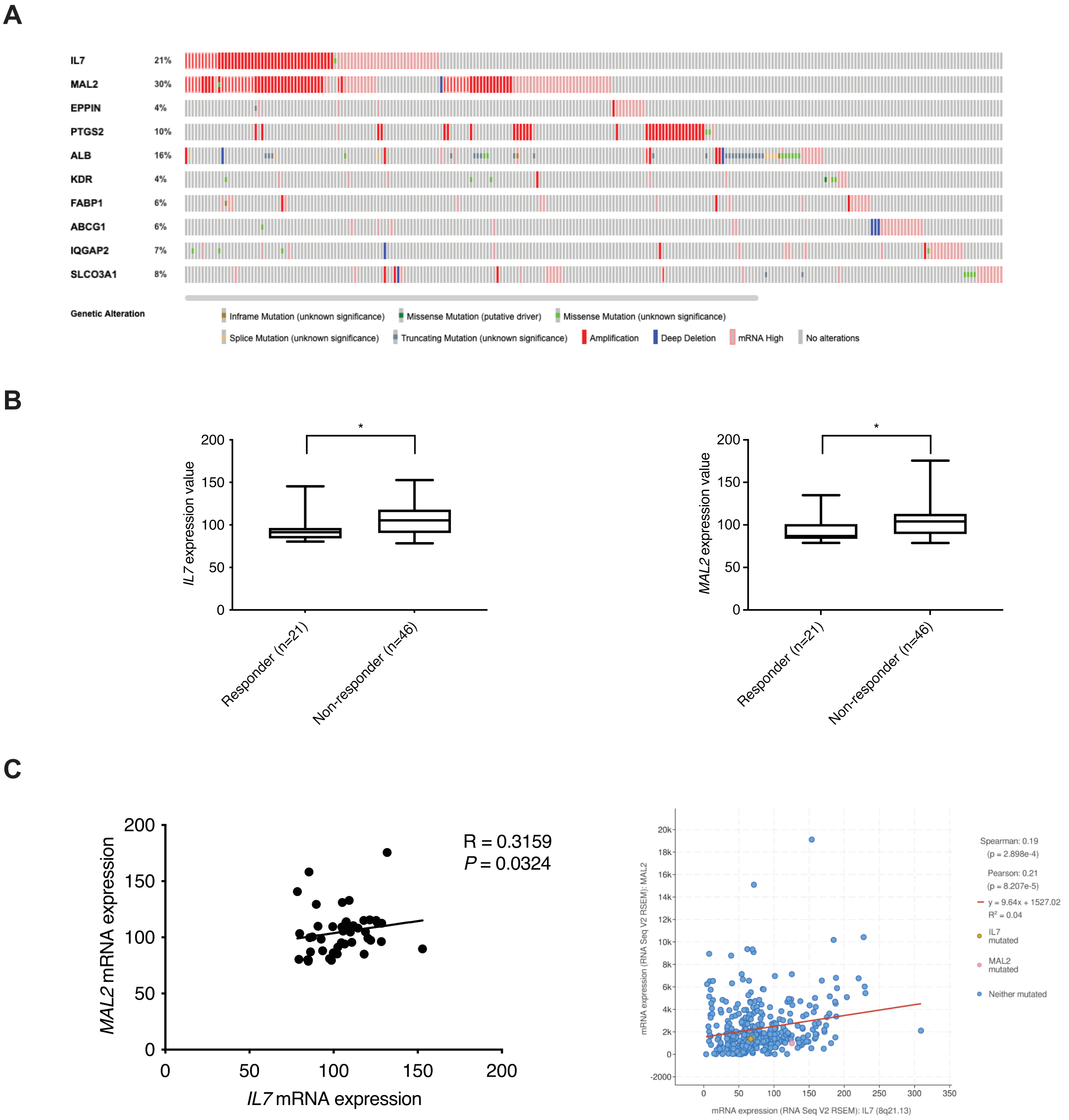

Supplementary Figure S2 Clinical relevance of *IL7* and *MAL2* as candidate genes in HCC-associated Sorafenib resistance. (**A**) *IL7* and *MAL2* were either amplified or showed high mRNA expression in majority of affected HCC patients. Co-occurrence of *IL7* and *MAL2* was found in the TCGA liver cancer database. (**B**) Expression of *IL7* and *MAL2* in Sorafenib non-responding patients (GSE109211) were significantly higher than responding patients. *, $P < 0.05$, two-tailed unpaired t-test. (**C**) Significant tendency for co-occurrence of *IL7* and *MAL2* was found in GSE109211 clinical study (**left**) and TCGA liver cancer database (**right**), based on their gene expression levels.
