# Supplementary material for: Sorafenib Resistance Contributed by IL7 and MAL2 in Hepatocellular Carcinoma Can Be Overcome by Autophagy-Inducing Stapled Peptides
Source: Cancers (Basel). 2023 Nov 3;15(21):5280. doi: 10.3390/cancers15215280 (PMC10650575; doi:10.3390/cancers15215280)

Figure 4A

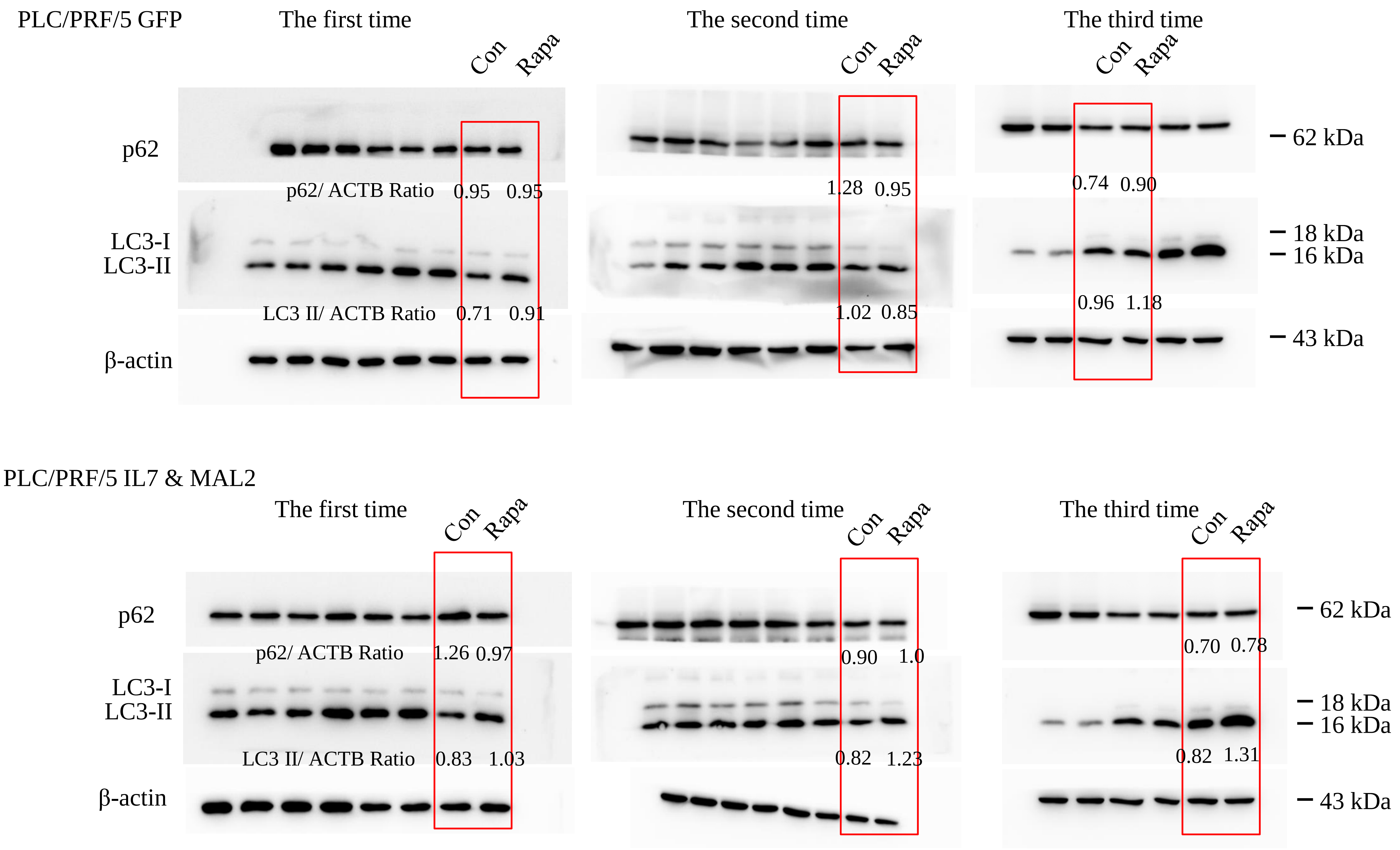

PLC/PRF/5 GFP
The first time
The second time
The third time
Con
Rapa
p62
p62/ ACTB Ratio
0.95 0.95
1.28 0.95
0.74 0.90
LC3-I
LC3-II
LC3 II/ ACTB Ratio
0.71 0.91
1.02 0.85
0.96 1.18
β-actin
62 kDa
18 kDa
16 kDa
43 kDa
PLC/PRF/5 IL7 & MAL2
The first time
The second time
The third time
Con
Rapa
p62
p62/ ACTB Ratio
1.26 0.97
0.90 1.0
0.70 0.78
LC3-I
LC3-II
LC3 II/ ACTB Ratio
0.83 1.03
0.82 1.23
0.82 1.31
β-actin
62 kDa
18 kDa
16 kDa
43 kDa

Figure 4C
PLC/PRF/5 GFP

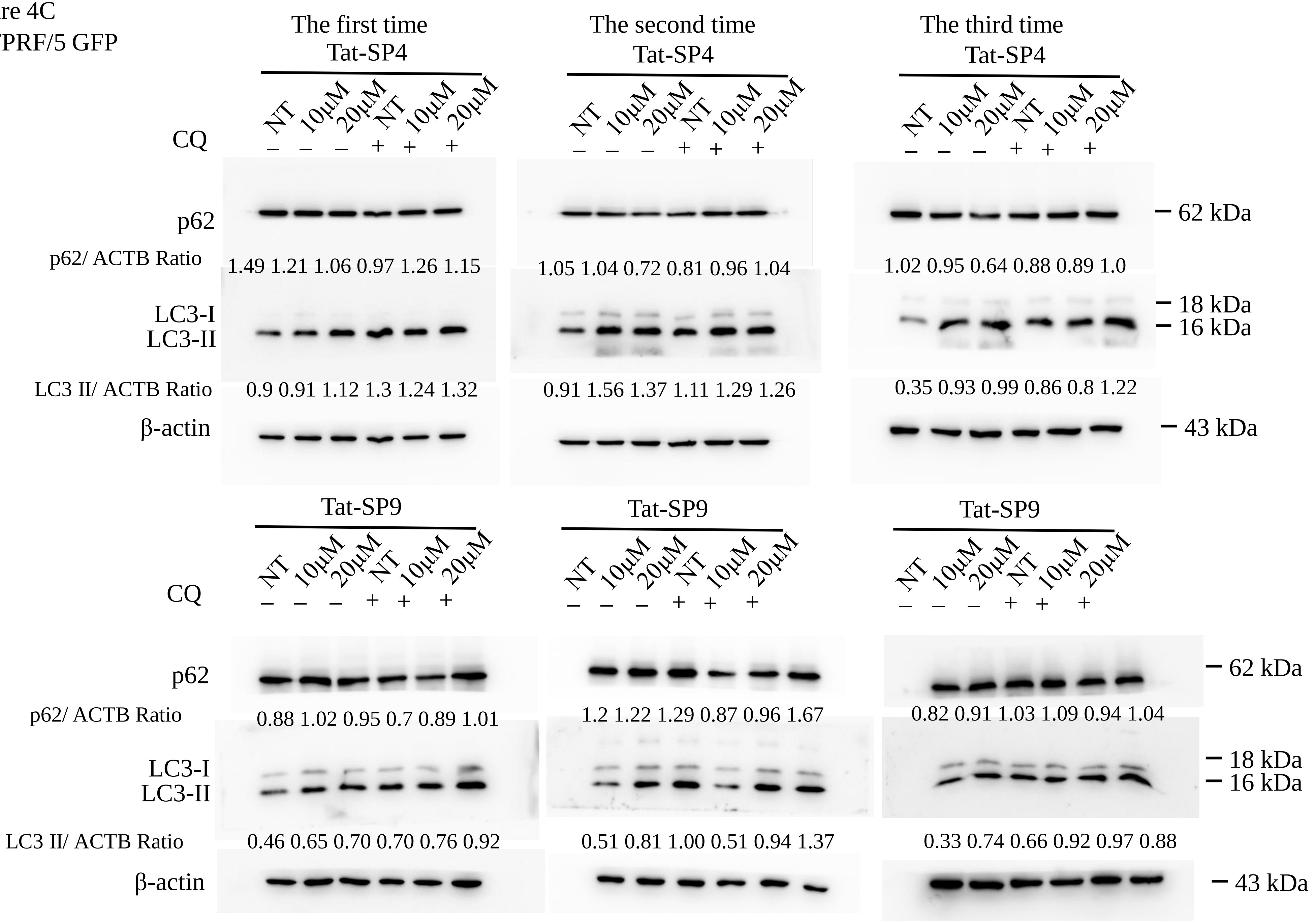

Figure 4E
PLC/PRF/5 IL7 & MAL2

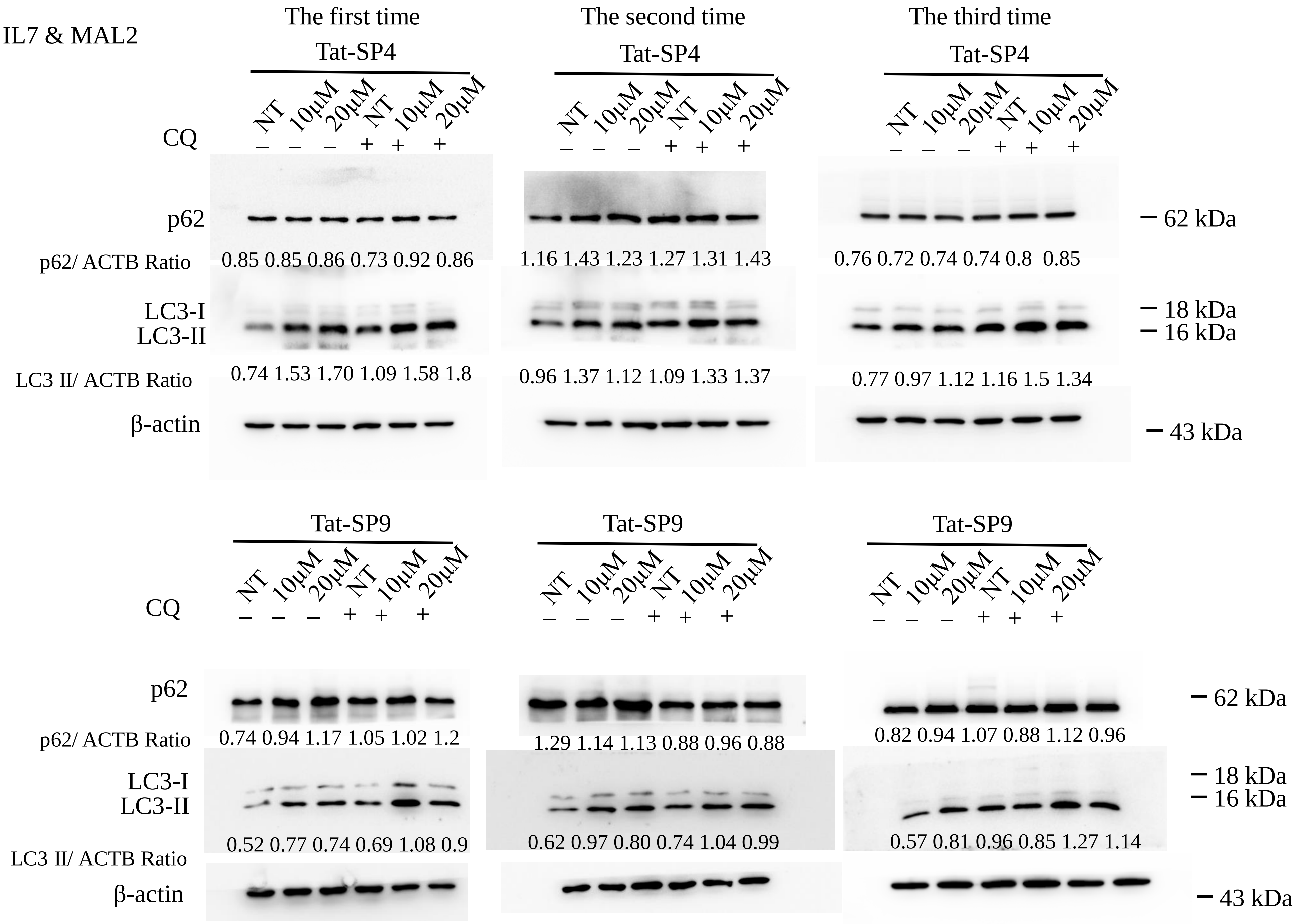

Supplement: Supplementary file 1 [file cancers-15-05280-s001.zip › WB Raw data of autophagy.pdf]
