# Supplementary figures and images for: Sorafenib Resistance Contributed by IL7 and MAL2 in Hepatocellular Carcinoma Can Be Overcome by Autophagy-Inducing Stapled Peptides
Source: Cancers (Basel). 2023 Nov 3;15(21):5280. doi: 10.3390/cancers15215280 (PMC10650575; doi:10.3390/cancers15215280)

40-

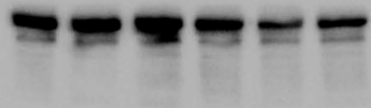

Supplement: Supplementary file 1 [file cancers-15-05280-s001.zip › ACTB_(JNK).pdf]

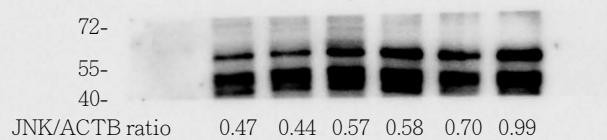

Supplement: Supplementary file 1 [file cancers-15-05280-s001.zip › JNK.pdf]

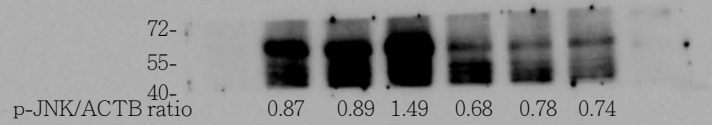

Supplement: Supplementary file 1 [file cancers-15-05280-s001.zip › P-JNK.pdf]

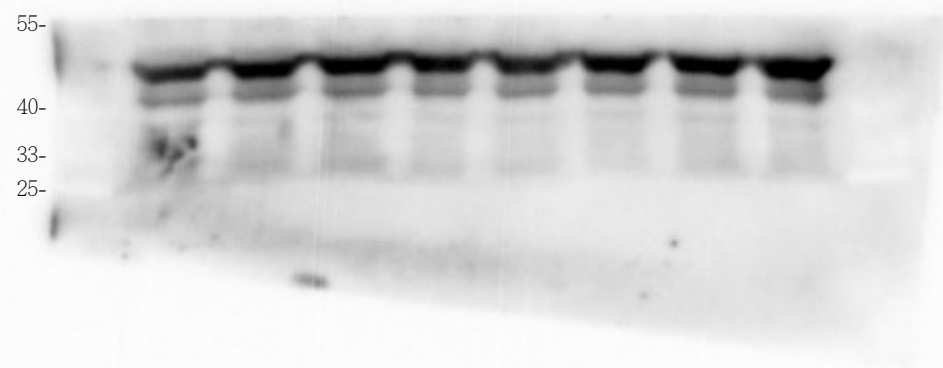

Supplement: Supplementary file 1 [file cancers-15-05280-s001.zip › PLC5_ACTB_(PI3K & AKT).pdf]

40-

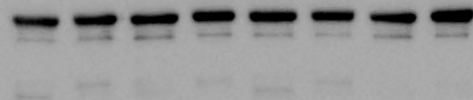

Supplement: Supplementary file 1 [file cancers-15-05280-s001.zip › PLC5_ACTB_(STAT3).pdf]

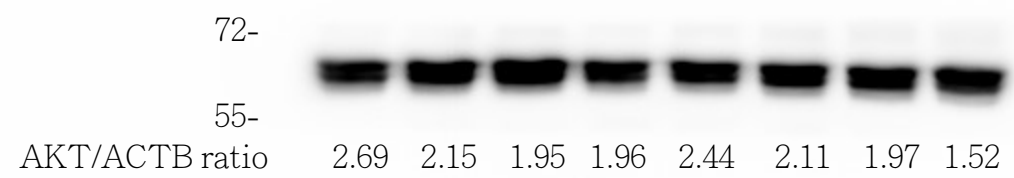

Supplement: Supplementary file 1 [file cancers-15-05280-s001.zip › PLC5_AKT_1.pdf]

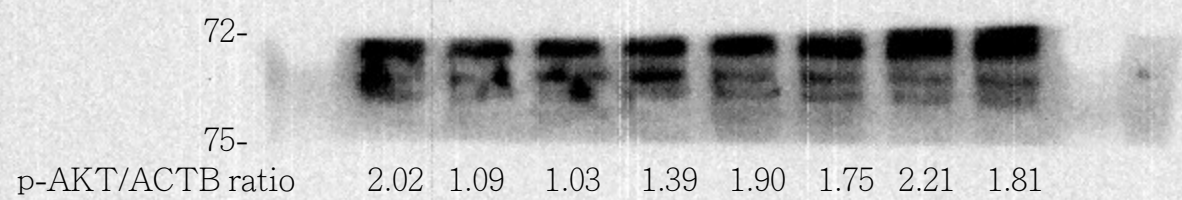

Supplement: Supplementary file 1 [file cancers-15-05280-s001.zip › PLC5_p-AKT_1.pdf]

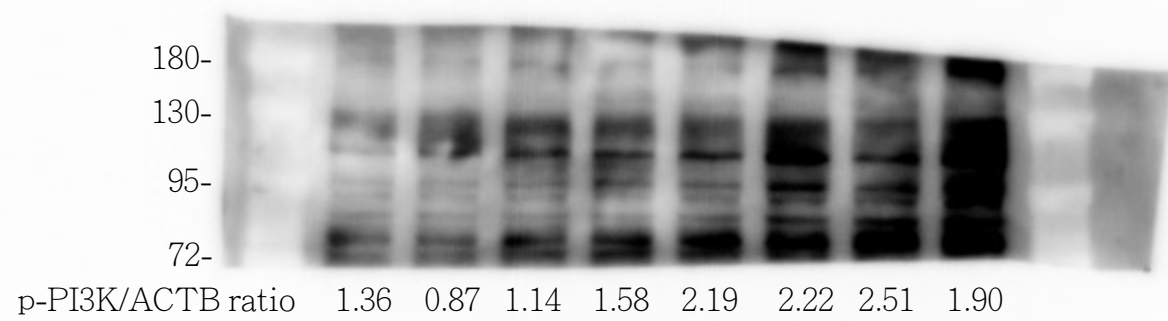

Supplement: Supplementary file 1 [file cancers-15-05280-s001.zip › PLC5_p-PI3K_1.pdf]

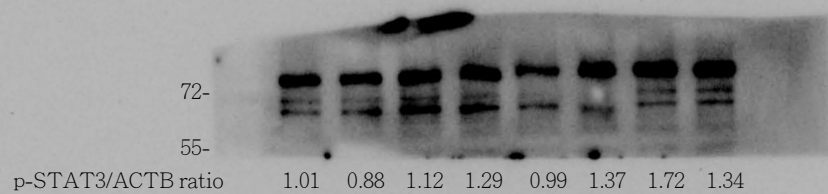

Supplement: Supplementary file 1 [file cancers-15-05280-s001.zip › PLC5_p-STAT3_1.pdf]

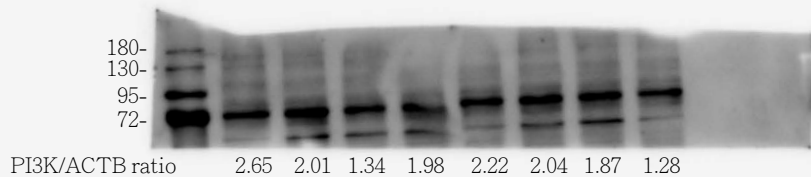

Supplement: Supplementary file 1 [file cancers-15-05280-s001.zip › PLC5_PI3K_1.pdf]

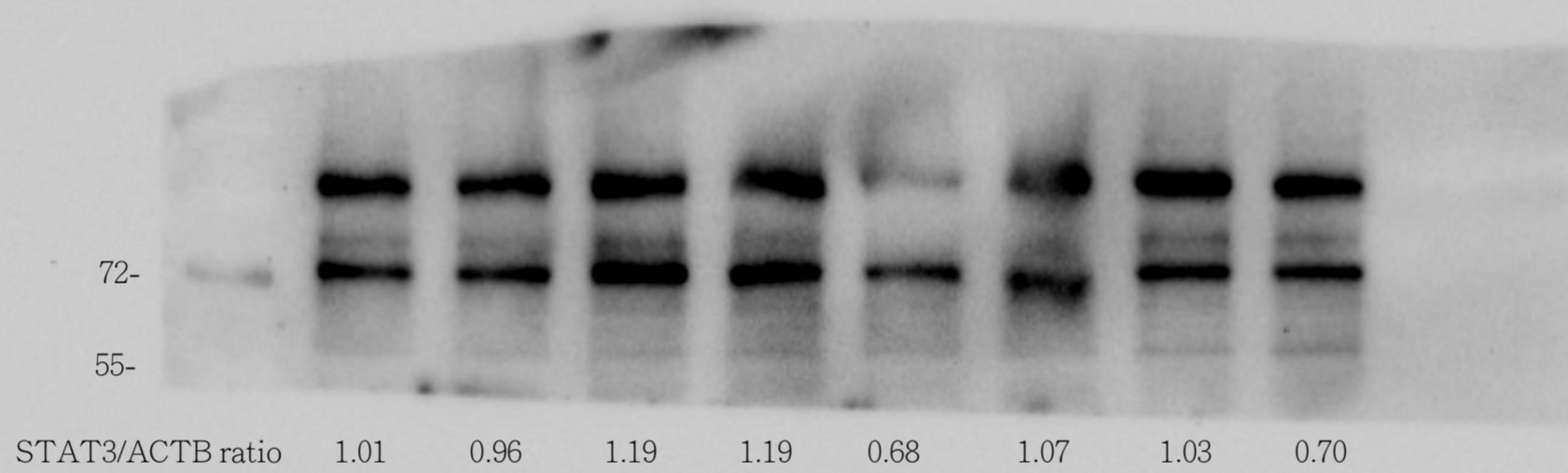

Supplement: Supplementary file 1 [file cancers-15-05280-s001.zip › PLC5_STAT3_1.pdf]
